# Supplementary material for: Controlling the bioactivity of a peptide hormone in vivo by reversible self-assembly
Source: Nat Commun. 2017 Oct 18;8:1026. doi: 10.1038/s41467-017-01114-1 (PMC5647335; doi:10.1038/s41467-017-01114-1)
Supplement: Supplementary file 1 — Supplementary Information [file 41467_2017_1114_MOESM1_ESM.pdf]

Supplementary Information

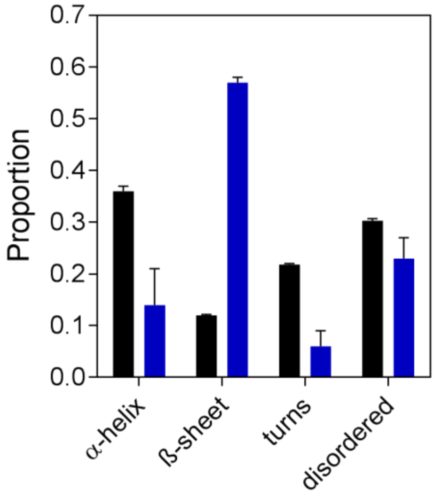

Supplementary Figure 1. Structural properties of free and fibrillar Oxm. Far-UV circular dichroism analysis of fibrillar (blue) and free (black) Oxm in 0.09% saline. The average ± s.d. of three samples is shown.

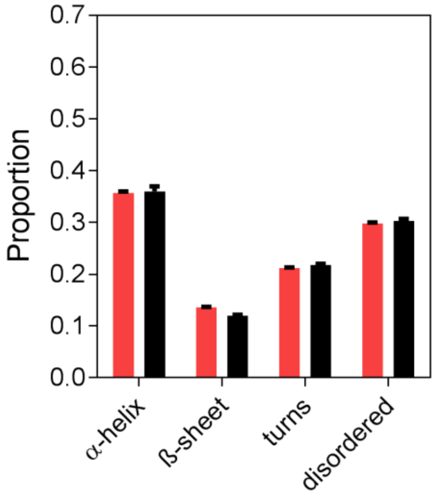

Supplementary Figure 2. Structural properties of free and released Oxm. Far-UV circular dichroism analysis of released (red) and free (black) Oxm in water. Oxm recovered its initial conformation, which is characterized by helical and disordered structures in proportions similar to free Oxm. The average ± s.d. of three samples is shown.

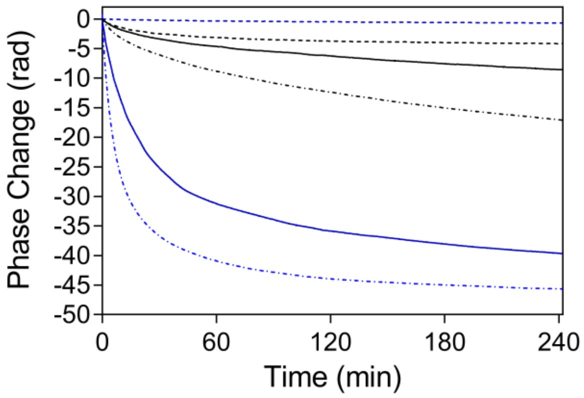

Supplementary Figure 3. Control experiments for material removal on top of the sensor chips. Phase changes as a function of time on top of the DPI sensor chips coated with collagen and fibrillar Oxm in water (blue solid line) or PBS (black solid line), water (blue dash-dot line) or TBS (black dash-dot line) and collagen in water (blue dashed line) or PBS (black dashed line).

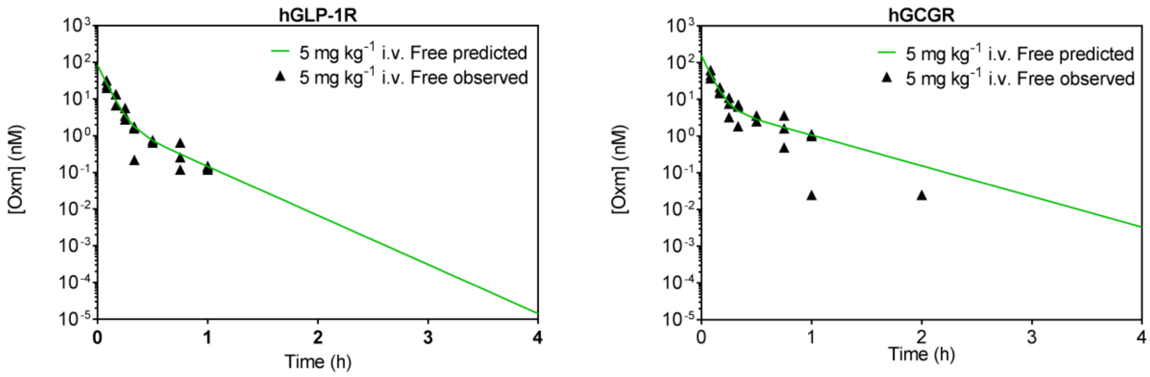

Supplementary Figure 4. hGLP-1R and hGCGR activity. Plots of observed vs. predicted hGLP-1R and hGCGR activity in rats treated with i.v.-administered free Oxm (5 mg kg^{-1}).

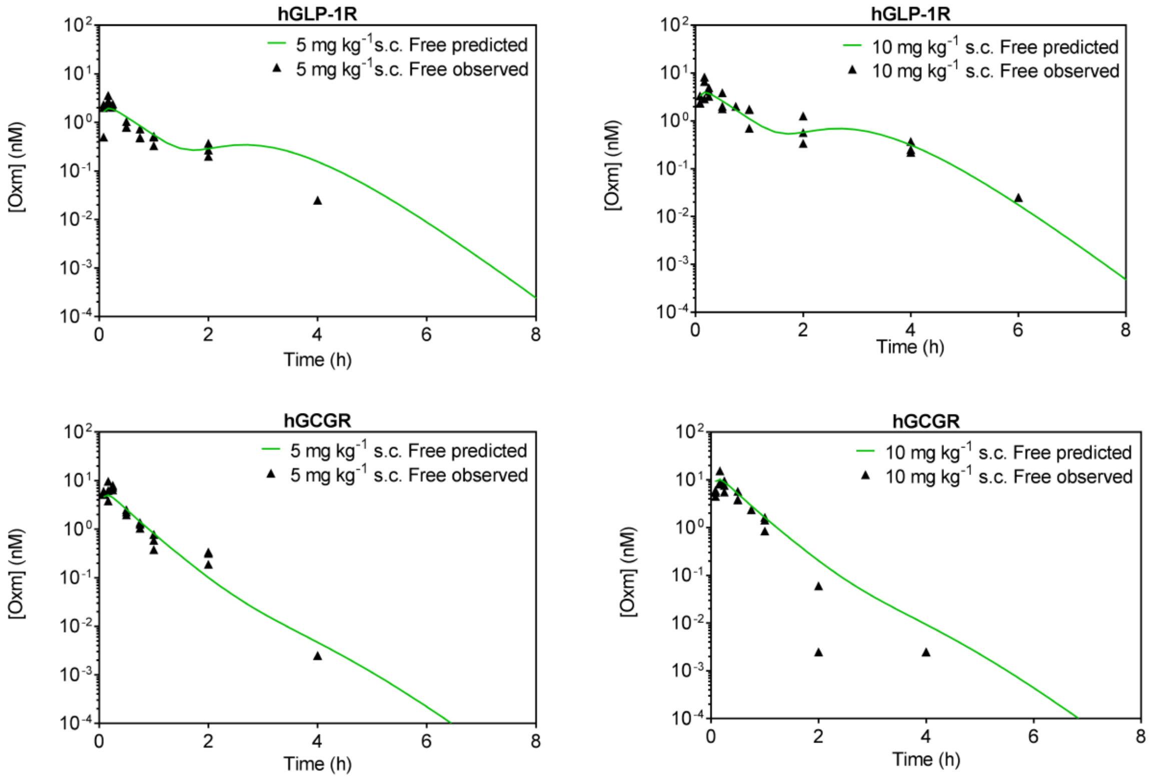

Supplementary Figure 5. hGLP-1R and hGCGR activity. Plots of observed vs. predicted hGLP-1R and hGCGR activity in rats dosed with 5 and 10 mg kg^{-1} s.c.-administered free Oxm.

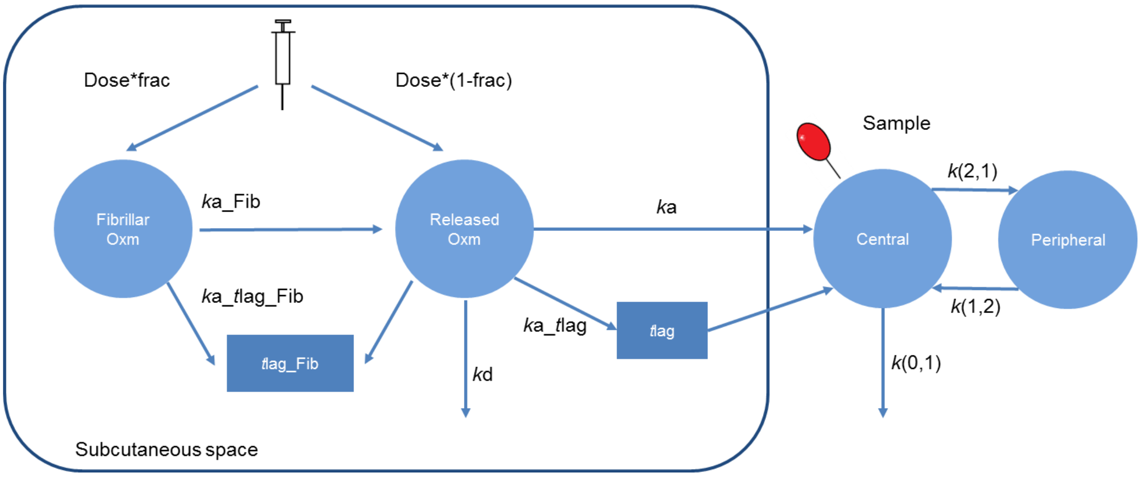

Supplementary Figure 6. **Schematic model of fibrillar Oxm pharmacokinetics**. Central = central compartment; peripheral = peripheral compartment; k_{a_Fib} = first-order absorption of fibrillar Oxm (h^{-1}); $k_{a\_tlag\_Fib}$ = transfer of fibrillar Oxm from the dosing to the lag compartment (h^{-1}); t_{lag_Fib} = lag time for fibrillar Oxm (h); k_a = first-order absorption of the s.c. depot (h^{-1}); k_{a_tlag} = transfer from the dosing to the lag compartment (h^{-1}); t_{lag} = lag time for fresh Oxm (h); k_d = irreversible loss from dosing compartment (h^{-1}); k(0,1) = irreversible loss from the central compartment (h^{-1}); k(1,2) = transfer from the peripheral to the central compartment (h^{-1}); k(2,1) = transfer from the central to the peripheral compartment (h^{-1}); dose = dose site; frac = fraction of dose of fibrillar Oxm available in the dosing compartment; dose*frac = fraction of dose delivered as fibrillar Oxm, dose*(1-frac) = fraction of dose delivered as free Oxm; sample = sampling site.

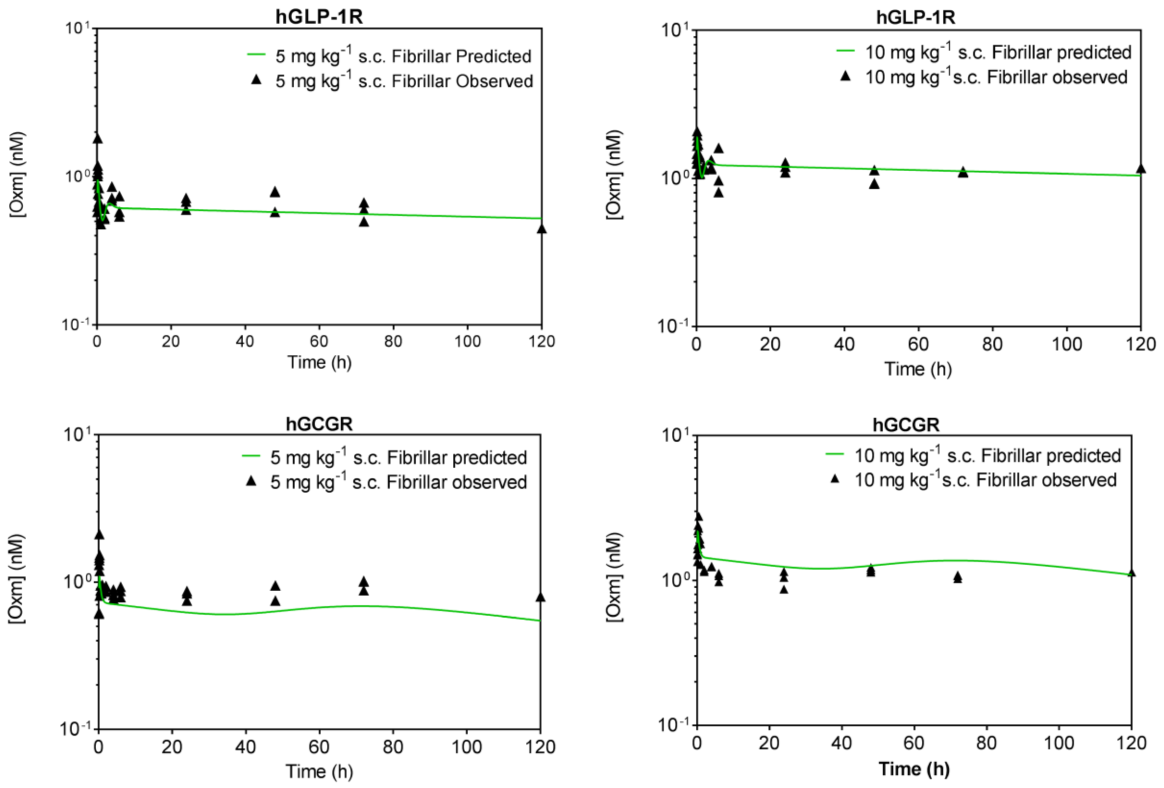

Supplementary Figure 7. hGLP-1R and hGCGR activity. Plots of observed vs. predicted hGLP-1R and hGCGR activity in rats dosed with 5 and 10 mg kg^{-1} s.c.-administered fibrillar Oxm.

% release

	saline	Phosphate buffer	Tris-HCl buffer	water	HCl
**4 h**	Not detected	Not detected	0.7±0.5	36.8±4.1<br>37.8±3.8[a]	76.8±1.7
**48 h**	Not detected	1.4±0.1	1.2±0.1	52.5±8.3	Not measured due to degradation

Supplementary Table 1. Stability properties of fibrillar Oxm. Release of peptide from 1 mg mL^{-1} fibrillar Oxm incubated in phosphate buffer (25 mM, pH 7.5), Tris-HCl buffer (25 mM, pH 7.5), saline (0.09%), water and HCl (10 mM, pH 2) at 37°C. The data shown correspond to the average ± s.d. of three different samples. [a]The data shown as mean ± s.e.m. from three independent batches of fibrillar Oxm and three samples from each batch.

Variation in % at 4 h

	**Δmass**	**Δthickness**	**Δdensity**
**PBS**	10.1±2.0	7.1±0.9	3.3±1.2
**Water**	42.3±4.6	39.7±5.6	4.4±4.0

Supplementary Table 2. Dissociation of fibrillar Oxm. Percent change in mass, thickness and density of fibrillar Oxm and collagen layers was measured by DPI after 4 h in water or PBS at 37°C. The data represent the average ± s.d. of three independent experiments.

	hGLP-1R EC_{50} (pM)		hGCGR EC_{50} (pM)	
	Geomean	s.e.m.	Geomean	s.e.m.
**GLP-1 (7–36 amide)**	0.8	0.2	NA	-
**Glucagon**	159.1	43.2	0.9	0.3
**Free Oxm**	72.4	17.9	18.2	4.2
**Fibrillar Oxm**	2938.3	655.7	1120.5	302.2
**Released Oxm**	96.6	31.0	26.0	5.2

Supplementary Table 3. Agonist potency. Summary of peptide potency in cAMP accumulation assay at hGLP-1R and hGCGR expressed in CHO cells. Data is geometric mean and standard error of mean from 5-7 independent experiments. NA = not active.

	**hGLP-1R**			**hGCGR**		
Parameter	Value	SD	CV	Value	SD	CV
V_c	$1.34\times10^{+01}$	$5.64\times10^{+00}$	(42)	$3.98\times10^{+00}$	$5.68\times10^{+00}$	(143)
$k(0,1)$	$1.28\times10^{+01}$	$2.61\times10^{+00}$	(20)	$1.80\times10^{+01}$	$1.51\times10^{+01}$	(84)
$k(1,2)$	$3.50\times10^{+00}$	$1.08\times10^{+00}$	(31)	$4.98\times10^{+00}$	$1.16\times10^{+00}$	(23)
$k(2,1)$	$1.36\times10^{+00}$	6.39×10^{-01}	(47)	$7.28\times10^{+00}$	$3.66\times10^{+00}$	(50)
alpha	$1.46\times10^{+01}$	$3.23\times10^{+00}$	(22)	$2.69\times10^{+01}$	$1.72\times10^{+01}$	(64)
beta	$3.07\times10^{+00}$	8.17×10^{-01}	(27)	$3.32\times10^{+00}$	1.85×10^{-01}	(6)
t_{half_alpha}	4.76×10^{-02}	1.05×10^{-02}	(22)	2.58×10^{-02}	1.65×10^{-02}	(64)
t_{half_beta}	2.26×10^{-01}	6.00×10^{-02}	(27)	2.08×10^{-01}	1.16×10^{-02}	(6)

Supplementary Table 4. Pharmacokinetic parameter estimates of i.v.-administered fresh Oxm. V_c = central volume of distribution (L kg^{-1}); $k(0,1)$ = elimination rate (h^{-1}); $k(1,2)$ = transfer rate from the peripheral to the central compartment (h^{-1}); $k(2,1)$ = transfer rate from the central to the peripheral compartment (h^{-1}). The parameters alpha and beta (h^{-1}) are the fast and slow exponents, respectively, and the distribution and elimination half-lives are reported as t_{half_alpha} and t_{half_beta} (h). hGLP-1R = parameter estimates based on fit to hGLP-1R activity assay, hGCGR = parameter estimates based on fit to hGCGR activity assay. SD = Standard Deviation, CV = Coefficient of Variation (%).

	hGLP-1R			hGCGR		
Parameter	Value	SD	CV	Value	SD	CV
k_a	4.42×10^{-01}	4.67×10^{-02}	(11)	8.10×10^{-01}	8.66×10^{-02}	(11)
k_{a_tlag}	2.11×10^{-01}	4.83×10^{-02}	(23)	1.02×10^{-01}	2.10×10^{-02}	(21)
k_d	$1.25\times10^{+00}$	1.40×10^{-01}	(11)	$3.40\times10^{+00}$	2.58×10^{-01}	(8)
t_{lag}	$2.45\times10^{+00}$	9.19×10^{-02}	(4)	$1.46\times10^{+00}$	4.66×10^{-02}	(3)
F	3.44×10^{-01}	2.43×10^{-02}	(7)	2.12×10^{-01}	1.26×10^{-02}	(6)
t_{half_ka}	$1.57\times10^{+00}$	1.66×10^{-01}	(11)	8.56×10^{-01}	9.16×10^{-02}	(11)
$t_{half\_ka\_tlag}$	$3.29\times10^{+00}$	7.54×10^{-01}	(23)	$6.78\times10^{+00}$	$1.39\times10^{+00}$	(21)

Supplementary Table 5. Pharmacokinetic parameter estimates of s.c.-administered fresh Oxm administered. F = bioavailability; k_a = first-order absorption rate (h^{-1}); k_{a_tlag} = transfer rate from the dosing to the lag compartment (h^{-1}); k_d = irreversible loss from the dosing compartment (h^{-1}); t_{lag} = lag time (h); t_{half_ka} = absorption half-life corresponding to the route without lag time (h); $t_{half\_ka\_tlag}$ = absorption half-life corresponding to the route with lag time. hGLP-1R = parameter estimates based on fit to hGLP-1R activity assay, hGCGR = parameter estimates based on fit to hGCGR activity assay. SD = Standard Deviation, CV = Coefficient of Variation (%).

	hGLP-1R			hGCGR		
Parameter	Value	SD	CV	Value	SD	CV
V_{c_Fib}	6.84×10^{-02}	4.43×10^{-02}	(65)	7.76×10^{-02}	2.80×10^{-02}	(36)
frac	9.98×10^{-01}	1.51×10^{-03}	(0.2)	9.97×10^{-01}	1.13×10^{-03}	(0.1)
k_{a_Fib}	1.01×10^{-03}	1.24×10^{-03}	(123)	4.50×10^{-03}	1.77×10^{-03}	(39)
$k_{a\_tlag\_Fib}$	3.88×10^{-04}	1.05×10^{-03}	(270)	2.60×10^{-03}	2.59×10^{-03}	(100)
t_{lag_Fib}	4.50×10^{-01}	8.78×10^{-01}	(195)	$5.64\times10^{+01}$	$1.07\times10^{+01}$	(19)
$t_{half\_ka\_Fib}$	$6.89\times10^{+02}$	$8.49\times10^{+02}$	(123)	$1.54\times10^{+02}$	$6.07\times10^{+01}$	(39)
$t_{half\_ka\_tlag\_Fib}$	$1.79\times10^{+03}$	$4.82\times10^{+03}$	(270)	$2.67\times10^{+02}$	$2.66\times10^{+02}$	(100)

Supplementary Table 6. Pharmacokinetic parameter estimates of s.c.-administered fibrillar Oxm. V_{c_Fib} = central volume of fresh Oxm after fibrillar Oxm administration (L kg^{-1}); frac = fraction of dose of fibrillar Oxm available in the dosing compartment; k_{a_Fib} = first-order absorption rate of fibrillar Oxm (h^{-1}); $k_{a\_tlag\_Fib}$ = transfer rate of fibrillar Oxm from the dosing to the lag compartment (h^{-1}); t_{lag_Fib} = lag time (h); $t_{half\_ka\_Fib}$ = absorption half-life of fibrillar Oxm corresponding to the route without lag time (h); $t_{half\_ka\_tlag\_Fib}$ = absorption half-life of fibrillar Oxm corresponding to the route with lag time (h). hGLP-1R = parameter estimates based on fit to hGLP-1R activity assay, hGCGR = parameter estimates based on fit to hGCGR activity assay. SD, standard deviation. CV = Coefficient of Variation (%).
